# Integrated metabolome and transcriptome analysis of differences in quality of ripe *Lycium barbarum* L. fruits harvested at different periods

**DOI:** 10.1186/s12870-024-04751-z

**Published:** 2024-02-02

**Authors:** Deshuai Liu, Miao Yuan, Ye Wang, Li Zhang, Wenkong Yao, Mei Feng

**Affiliations:** 1 College of Enology and Horticulture, Ningxia University, Yinchuan, 750021 Ningxia China; 2 Ningxia Modern Facility Horticulture Engineering Technology Research Center, Yinchuan, 750021 Ningxia China; 3 Ningxia Key Laboratory of Modern Molecular Breeding of Dominant and Characteristic Crops, Yinchuan, 750021 Ningxia China

**Keywords:** Wolfberry, Fruit quality, Picking time, Abscisic acid, Glutamic acid, Ascorbate–glutathione recycling system

## Abstract

**Background:**

Wolfberry is well-known for its high nutritional value and medicinal benefits. Due to the continuous ripening nature of Goji berries and the fact that they can be commercially harvested within a few weeks, their phytochemical composition may change during the harvesting process at different periods.

**Result:**

The involved molecular mechanisms of difference in fruit quality of ripe *Lycium barbarum* L. harvested at four different periods were investigated by transcriptomic and metabolomics analyses for the first time. According to the results we obtained, it was found that the appearance quality of *L. barbarum* fruits picked at the beginning of the harvesting season was superior, while the accumulation of sugar substances in *L. barbarum* fruits picked at the end of the harvesting season was better. At the same time the vitamin C and carotenoids content of wolfberry fruits picked during the summer harvesting season were richer. Ascorbic acid, succinic acid, glutamic acid, and phenolic acids have significant changes in transcription and metabolism levels. Through the network metabolic map, we found that ascorbic acid, glutamic acid, glutamine and related enzyme genes were differentially accumulated and expressed in wolfberry fruits at different harvesting periods. Nevertheless, these metabolites played important roles in the ascorbate–glutathione recycling system. Ascorbic acid, phenolic substances and the ascorbate–glutathione recycling system have antioxidant effects, which makes the* L. barbarum* fruits harvested in the summer more in line with market demand and health care concepts.

**Conclusion:**

This study laid the foundation for understanding the molecular regulatory mechanisms of quality differences of ripe wolfberry fruits harvested at different periods, and provides a theoretical basis for enhancing the quality of* L. barbarum* fruits.

**Supplementary Information:**

The online version contains supplementary material available at 10.1186/s12870-024-04751-z.

## Background

The genus *Lycium* belongs to Solanaceae family and includes about 97 species, which are widely distributed in temperate and subtropical zones [1]. They are a perennial deciduous shrub mainly distributed in southeastern Europe, northwestern China and the Mediterranean region [2]. In China, the genus *Lycium* has seven species and two varieties, of which *Lycium barbarum* L. and *L. chinense* Mill. have been transformed into globally traded commodities [3]. The fruit of *L. barbarum* and *L. chinense*, which are commonly known as wolfberry, Goji berry or Goji, and have been used as a traditional herbal medicine and food additive in China for more than 2000 years [4, 5]. It is popular worldwide because it is promoted in numerous media as a healthful and anti-aging food [6]. In traditional Chinese medicine wolfberry was considered to have the function of nourishing the liver and kidney, and brightening the eyes [7]. Modern pharmacological studies have shown that wolfberry has the ability to improve vision, enhance immunity, lower blood sugar and lipid levels, reduce the risk of cancer, neuroprotection, antioxidant and anti-aging effects [7–9].

Research on fruit quality not only helps us to select breeding programs by exploring the most promising fruit genotypes, but also helps us to find the method for improving fruit quality, exploring the health-promoting components and market value [10, 11]. Nowadays, with the improvement of people’s living standards and the enhancement of health awareness, the market demand for wolfberry has increased [12]. People’s consumption pattern is gradually changing from dried fruits to fresh fruits, and fresh Goji berries will gradually become the first choice [13]. Wolfberry has been recognized as the latest “super fruit” in world, and its fruit is rich in polysaccharides, polyphenols, flavonoids, carotenoids, betaine, amino acids, organic acids, vitamins and minerals, and other bioactive substances [14–16]. The content of these nutrients in berries is an important factor in determining the sensory and nutritional quality of berries, and is the main indicator for evaluating the quality of fruit [17]. Total sugars, total soluble solids and titratable acids are also commonly used to assess berry quality [18]. Fruit appearance quality (fruit length, fruit width, fruit weight, fruit color) is also an important characteristic that affects its exploitation and commercial value. The contents of carotenoids in the fruits harvested in summer were higher than those harvested in autumn, and the appearance quality was better [19]. By comparing the berries harvested in four periods from June to September, it was found that the berries harvested in June had the greatest length, width and weight and the best appearance quality [20]. The comparative analysis of the physicochemical indexes of the berries from four different harvesting periods, the results showed that the fruit appearance quality was better at the beginning of the production season, while the nutritional quality was better at the end of the harvesting season. This means that fruits harvested earlier are more suitable for fresh consumption, while fruits harvested later are more suitable for industrial processing [21].

Although the *L. barbarum* is distributed around the world, but in northwest China, Ningxia Hui Autonomous Region has become the main production area of wolfberry. Ningxia has a semi-arid climate, suitable cultivation conditions, a long history of cultivation, and especially a reputation for producing high quality wolfberries [22]. At present, there are many reports on the changes and accumulation of nutrients in different developmental stages and varieties of *L. barbarum* [18, 23, 24]. However, only few studies have investigated the molecular mechanisms underlying the differences in quality of ripe wolfberry fruits harvested at different periods. The wolfberry variety ‘Ningqi 1’ is the main commercial cultivar of *L. barbarum*. It has the largest planting area and accounts for more than 80% of China’s total yield [25]. Therefore, in this study, we selected ripe *L. barbarum* fruits harvested at four different periods of ‘Ningqi 1’ as the research object to investigate the quality differences of *L. barbarum* fruits harvested at different periods. RNA sequencing (RNA-Seq) and ultra-performance liquid chromatography tandem mass spectrometry (UPLC-MS) were used for transcriptome sequencing and untargeted metabolomic analysis of *L. barbarum* fruits harvested at four different periods. Integrative analysis of transcriptomics and metabolomics was performed to reveal the regulatory mechanisms of fruit quality differences in ripe *L. barbarum* harvested at different periods, providing a theoretical basis for enhancing the quality of Goji berry fruits.

## Materials and methods

### Plant materials and sample collection

*L. barbarum* is a deciduous shrub of the Solanaceae family, growing 0.8–2 m high and stem tufts with short spines. The leaves are lanceolate to ovate. There are 1 to 3 axillary, radical flowers, pedicel is 1–2 cm long, calyx usually has 2 lobes that are 2-ribbed or 3-ribbed at the end. The corolla is funnel-shaped, light purple or violet with a 5-lobed margin. The corolla tube is 8–10 mm long and longer than the lobes [26]. The berry is fusiform or oblong shaped with acute apex, 6–20 mm in length, 3–8 mm in diameter, pericarp orange or dark red. The pulp is fleshy and soft with a bitter and sweet taste [4, 26]. Wolfberry has a long flowering and fruiting period, generally from May to September can be continuous blooming and bearing fruit, the fruit is mainly harvested in summer to autumn. Goji berries can ripen continuously, and picking of ripe berries is done between June and September. Ripe fruits are commercially harvested approximately every 10–15 days, and fruits are harvested 2–3 times in a month [20, 21].

**Fig. 1:**
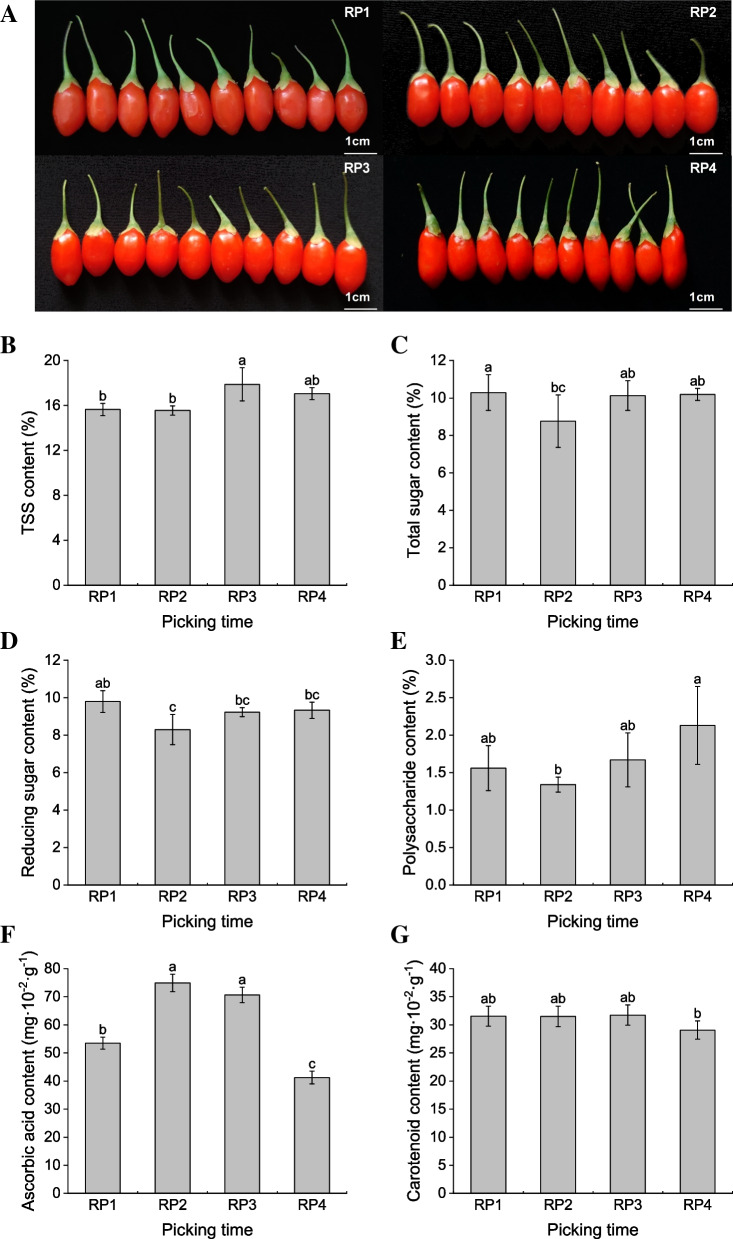
Changes in nutrient indexes of ripe *L. barbarum* fruits harvested at four different periods.** A** Ripe fruit samples of *L. barbarum* collected at four different periods.** B** Content of total soluble solids (TSS). **C** Content of total sugar. **D** Content of reducing sugar. **E** Content of polysaccharide. **F** Content of ascorbic acid. **G** Content of carotenoid. Statistical significance was determined by ANOVA and Duncan’s test post hoc analysis. Different lowercase letters indicate significant differences between treatments (*P* < 0.05)

The 15-year-old Ningxia *L. barbarum* ‘Ningqi 1’ was cultivated with row spacing of 3.0 × 1.0 m and plant height of 1.5 m in Yinchuan City, Ningxia Hui Autonomous Region, China (106°6′56″E, 38°38′20″N; elevation = 1082 m). All experimental plants were 15 years old and the plants were grown under the same conditions of soil, fertilizer and water management. In this study, ripe *L. barbarum* fruits were picked on June 17 (RP1, first ripening period), July 3 (RP2, second ripening period), July 19 (RP3, third ripening period) and September 20 (RP4, fourth ripening period), 2021 were used as research materials (Fig. 1A). All fruits were harvested from the same orchard area. The collected fruit without decay, mold and mechanical damage were wrapped in tin foil and frozen in liquid nitrogen and stored at -80℃ for subsequent use. All sample materials were collected between 9 and 10 am from the same side of the tree, on the same branch. At each sampling time point, ripe berries were collected from six wolfberry trees by one person. Three biological replicates of RNA preparation six biological replicates of metabolite extraction were used in this study [27].

### Determination of physicochemical indexes of *L. barbarum* fruits

One hundred fruit samples were randomly selected by quartering method and weighed to 0.01 g precision. Total soluble solid (TSS) content was measured using a handheld refractometer (Atago Co., Ltd, Tokyo, Japan) according to the method described by Zhao et al. [28]. The extraction and determination of *L. barbarum* polysaccharide (LBP) were conducted with reference to the methods in Chinese Pharmacopoeia and national standard (GB/T 18672–2014). The contents of reducing sugar and total soluble sugar in wolfberry fruits were determined according to the method of plant reducing sugar and total soluble sugar content kit (Suzhou Keming Biotechnology Co., Ltd, Jiangsu, China). The content of vitamin C in wolfberry fruits was determined by using ascorbic acid (AsA) content kit (Suzhou Gerisi Biotechnology Co., Ltd, Jiangsu, China). Determination of carotenoid content in wolfberry fruits by using plant carotenoid content detection kit (Solarbio Science & Technology Co., Ltd, Beijing, China).

### RNA sequencing data analysis

Total RNA was extracted from the fruit samples using pre-frozen TRIzol reagent, and then the quantity and quality of total RNA was extracted and assessed using NanoDrop 2000 (Nanodrop Thermo Scientific, Wilmington, DE, USA) and Agilent Bioanalyzer 2100 (Agilent Technologies, Santa Clara, CA, USA). The Illumina NovaSeq platform (Personal Biotechnology Co., Ltd, Shanghai, China) was used to screen samples with RNA integrity number (RIN) > 8.0 for next-generation sequencing (NGS) analysis [29]. The clean reads were then mapped to the reference genome (GCA_019175385.1_ASM1917538v1_genomic.fna) [30], using the HISAT2 software. Gene expression was normalized to Fragments Per Kilo bases per Million fragments (FPKM) using HTSeq. Differential analysis of gene expression was performed using DESeq with a threshold of |log2FoldChange|> 1 for differentially expressed genes (DEGs) and a significance *P*-value < 0.05 [31]. Cluster analysis of DEGs using R language Pheatmap software package, the volcano map of DEGs was drawn by using the R language ggplots2 software package [29, 32]. GO (Gene Ontology, http://geneontology.org/) enrichment and KEGG (Kyoto Encyclopedia of Genes and Genomes, www.kegg.jp/kegg/kegg1.html) were performed using the topGO and clusterprofiler R packages, respectively, with a significance *P*-value < 0.05 [33–35].

### Metabolites extraction and UPLC‑MS analysis

The fruit samples stored at -80℃ were thawed slowly in a refrigerator at 4℃, and then an appropriate amount of sample was added to a pre-chilled methanol–acetonitrile-water (2:2:1, v/v) solution, mixed by vortexing and sonicated at low temperature for 30 min. Then samples were left at -20℃ for 10 min and centrifuged at 14,000 g for 20 min at 4℃, and the supernatant was dried under vacuum, and 100 μL of aqueous acetonitrile solution (acetonitrile:water = 1:1, v/v) was added, after the vortexing and centrifuging at 14,000 g for 15 min at 4℃. the sample extracts were separated on an ultra-performance liquid chromatography (UPLC, Agilent 1290, Agilent Technologies, CA, USA) system with an ACQUITY UPLC BEH C_18_ (100 mm × 2.1 mm, 1.7 μm, Waters, Milford, USA) at a column temperature of 40℃, flow rate of 0.4 mL·min^−1^; injection volume of 2 μL, mobile phase A (water containing 25 mM ammonium acetate and 0.5% formic acid, and mobile phase B (methanol). The gradient elution conditions were performed as follows: 0 ~ 0.5 min, 5% B; 0.5 ~ 10 min, B varied linearly from 5 to 100%; 10.0 ~ 12.0 min, B maintained at 100%; 12.0 ~ 12.1 min, B varied linearly from 100 to 5%; 12.1 ~ 16 min B was maintained at 5%. The samples were then acquired by primary and secondary spectra using an AB Triple TOF 6600 mass spectrometer (AB SCIEX, Framingham, MA, USA).

### Metabolomic data analysis

Unsupervised principal component analysis (PCA) and supervised orthogonal partial least squares discrimination analysis (OPLS-DA) were used to reflect intra group and inter group variability. The Student’s *t*-test and variable importance for the projection (VIP) obtained from the OPLS-DA model were used to identify metabolites that differed significantly between groups. The VIP > 1 and *P*-value < 0.05 were used as thresholds to screen out differential accumulation metabolites (DAMs) [36]. The screened differential metabolites were subjected to biological information mining by advanced analytical tools such as cluster analysis, correlation analysis, and KEGG (Kyoto Encyclopedia of Genes and Genomes, www.kegg.jp/kegg/kegg1.html) pathway annotation and analysis [33–35].

### Integrative analysis of transcriptome and metabolome

Integrative analysis of metabolome and transcriptome was performed on ripe wolfberry fruits harvested at four different periods. Differentially expressed metabolites and genes were first obtained from the results of quantitative assay analysis of the metabolome and transcriptome. The genes encoding the relevant enzymes were then obtained based on the metabolite information in the KEGG database (www.kegg.jp/kegg/kegg1.html) [33–35]. The differentially expressed metabolites and genes obtained above were mapped to the KEGG pathway database (www.kegg.jp/kegg/kegg1.html) to identify the relevant KEGG pathways at both transcriptional and metabolomic levels [33–35]. For genes encoding related enzymes, the homologous gene KO (KEGG Orthology) number, gene name, and enzyme EC (Enzyme Commission) number of the metabolic pathway involved can be obtained in the KEGG homologous gene database (www.kegg.jp/kegg/ko.html) [33–35]. For differential accumulation metabolites mapped to relevant metabolic pathways compound ID, compound name and metabolic pathway involved can be obtained in the KEGG small molecule database (www.kegg.jp/dbget-bin/www_bfind?compound) [29].

### Verification of RNA-Seq data and enzyme activity assays

Nine DEGs involved in lipid, carbohydrate and amino acid metabolic pathways were validated by quantitative real-time PCR (qRT-PCR). Designing specific primers for DEGs using Primer Premier 5.0 software (Primer, Canada). The *LbRH52* was used as an internal reference gene to determine the relative expression level of DEGs [37]. The sequences of the primers used for qRT-PCR are listed in Supplementary Table S1. The SYBR® *Premix Ex Taq*™ kit (TaKaRa, Japan) was used for the qRT-PCR reaction. The qRT-PCR reaction system was 20 µL, as follows: 1μL upstream primer and downstream primer, 1 μL cDNA template, 10 μL SYBR reagent and7 μL ddH_2_O. The PCR program was as follows: pre-denaturation at 95℃ for 10 min, followed by 40 cycles of 95 °C for 15 s, 60℃ for 30 s. Three biological replicates were set up for each sample, and the relative expression of genes was calculated using the 2^−∆∆CT^ method [38]. Measurement of glutamic acid and glutamine content in Goji berries using high-performance liquid chromatography according to the method described by Zhao et al. [28]. Gal LDH, APX, AAO, MDHAR, GOGAT, GS enzyme activities were measured separately using enzyme activity assay kits (Suzhou Keming Biotechnology Co., Ltd, Jiangsu, China).

### Statistical analysis

We used three biological and three technical replicates for all the measurements, which are reported as mean values in the results and mean values ± SD in the figures and tables [23]. Statistical significance was calculated by analysis of variance (ANOVA) and Duncan’s test, conducted using IBM SPSS 25.0 software (SPSS Inc., Chicago, IL, USA). In all data analyses, the significance level was* P* < 0.05.

## Results

### Analysis of appearance and nutritional quality of *L. barbarum* fruits harvested at four different periods

The appearance quality and nutritional quality of berries are significant factors affecting the cultivation and market consumption of berry crops [20]. Goji berries have the botanical characteristics of continuous flowering and fruiting, and their appearance and nutritional quality may potentially change during continuous commercial harvesting. The highest 100-grain fresh weight of 69.34 g was recorded for berries picked on June 17. In terms of fresh weight/dry weight, the highest fruit was harvested on June 17. The fruits picked on July 19 had the highest 100-grain dry weight of 17.44 g. The 100-grain fresh weight, 100-grain dry weight and fresh weigh/dry weight of wolfberry harvested on September 20 were the lowest (Table 1). Based on the results obtained, it was found that the100-grain fresh weight, 100-grain dry weight and fresh weigh/dry weight of wolfberry fruits harvested in the summer were generally higher than those of autumn fruits, with RP1(first ripening period) picked at the beginning of the harvesting season having relatively better appearance quality.

**Table 1:** Variation in weight of ripe *L.barbarum* fruits harvested at four different periods

Picking time (No.)	100-grain fresh weight (g)	100-grain dry weight (g)	Fresh weight/Dry weight
June 17th (RP1)	69.34 ± 2.67a	15.84 ± 0.94bc	4.31 ± 0.13a
July 3rd (RP2)	66.83 ± 3.12ab	16.19 ± 0.82b	4.17 ± 0.15a
July 19th (RP3)	68.29 ± 3.48ab	17.44 ± 0.94a	3.92 ± 0.24b
September 20th (RP4)	56.48 ± 2.39c	15.02 ± 0.80c	3.77 ± 0.13b

Statistical significance was determined by ANOVA and Duncan’s test post hoc analysis. Different lowercase letters indicate significant differences between treatments (*P* < 0.05), and the values shown are the means ± SD

The chemical composition content of *L. barbarum* fruits harvested at four different periods was analyzed. The results showed that RP1 had the highest content of total and reducing sugars (Fig. 1C, D). The highest ascorbic acid (also called vitamin C) content was found in RP2 (second ripening period) reaching of 74.94 mg∙10^–2^∙g^−1^, and followed by RP3 (third ripening period) reaching of 70.66 mg∙10^–2^∙g^−1^ (Fig. 1F). The contents of total soluble solids (TSS) and carotenoids were highest in RP3 (Fig. 1B, G). The highest polysaccharide content in RP4 (fourth ripening period) was 2.03% (Fig. 1E). Through a comprehensive analysis of six evaluation indicators, it was found that RP4 harvested in autumn has an advantage in sugar accumulation, while RP1, RP2, and RP3 harvested in summer perform better in terms of vitamin C and carotenoid content.

### Transcriptome sequencing analysis of *L. barbarum* fruits harvested at four different periods

**Fig. 2:**
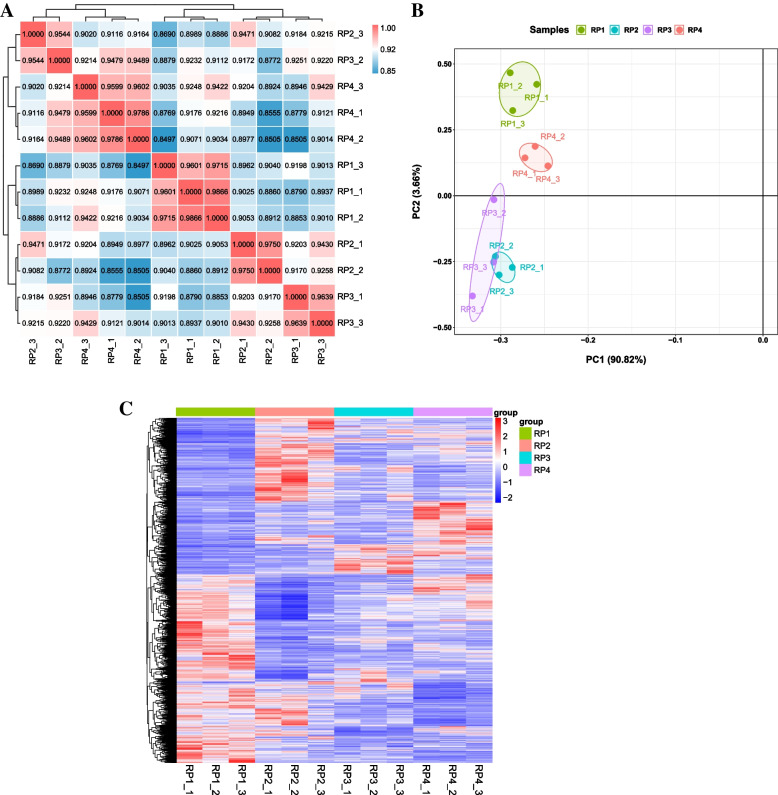
Transcriptomic analysis of ripe *L. barbarum* fruits harvested at four different periods. **A** Correlation analysis of gene expression levels between samples. **B** Principal component analysis of gene expression in each sample. The *X*-axis represents the first principal component and the *Y*-axis represents the second principal component. The same shape represents biological replicates of the same sample, different shapes represent different samples. **C** Cluster heat map analysis of DEGs among the different samples

A total of 12 samples of ripe *L. barbarum* fruit harvested at four different periods from ‘Ningqi 1’ were sequenced using the Illumina NovaSeq sequencing platform. A total of 83.16 Gb of raw reads were generated from transcriptome sequencing, and 76.29 Gb of clean reads were obtained by removing reads with connectors and low quality. The clean reads of each sample reached more than 5.43 Gb, and the Q20 and Q30 of clean reads exceeded 97% and 93%, respectively (Table 2). The average mapping rate of these clean reads on the *L. barbarum* reference genome was 94.39%, of which more than 96.18% reads were uniquely mapped to the reference genome, and more than 92.51% of the reads were mapped to exon regions (Table S2). This indicated that the reference genome was selected appropriately and the experimental accuracy was appropriate for subsequent analysis. The correlation coefficients (R^2^) of the three biological replicates of fruit samples from the four periods were greater than 0.9, indicating that the sample selection was reasonable and the transcriptome data had had high repeatability (Fig. 2A). Principal component analysis (PCA) showed that two principal components (PC1, PC2) were able to distinguish fruit samples collected at four different periods, with PC1 and PC2 explaining 90.82% and 3.66% of the variation, respectively. This was able to reflect significant changes in the transcriptional level of genes in the berries of the four periods (Fig. 2B). The subsequent clustering analysis also indicated that the expression of RP1, RP2, RP3 and RP4 gene clusters could be distinguished. This clustering pattern indicated that there were significant differences in the expression levels of genes in Goji berries harvested at different times (Fig. 2C).

**Table 2:** Features and quality of RNA-Seq raw data

Sample	Raw reads	Raw bases	Clean reads	Clean bases	Valid bases (%)	Q20 (%)	Q30 (%)
RP1_1	45,914,534	6,895,646,368	41,726,616	6,266,750,510	90.87	97.63	93.56
RP1_2	48,036,148	7,217,616,994	44,572,134	6,697,170,792	92.78	97.63	93.49
RP1_3	47,829,164	7,186,213,058	44,149,200	6,633,354,482	92.3	97.51	93.30
RP2_1	46,457,352	6,980,322,962	42,578,012	6,397,508,672	91.65	97.71	93.75
RP2_2	47,470,350	7,132,904,362	44,248,692	6,648,837,450	93.21	97.54	93.33
RP2_3	48,546,582	7,298,915,562	45,014,520	6,767,918,172	92.72	97.62	93.48
RP3_1	48,015,086	7,214,447,948	44,487,068	6,684,386,548	92.65	97.74	93.80
RP3_2	47,558,692	7,146,123,968	44,003,958	6,612,037,640	92.52	97.71	93.75
RP3_3	46,028,504	6,916,369,072	41,852,288	6,288,926,616	90.92	97.79	93.93
RP4_1	44,978,712	6,781,963,606	39,775,698	5,997,432,980	88.43	97.90	94.11
RP4_2	42,613,496	6,434,637,896	38,825,710	5,862,682,210	91.11	97.98	94.30
RP4_3	39,436,484	5,954,909,084	35,960,550	5,430,043,050	91.18	98.07	94.41

### Differential expression analysis of genes in *L. barbarum* fruits harvested at four different periods

**Fig. 3:**
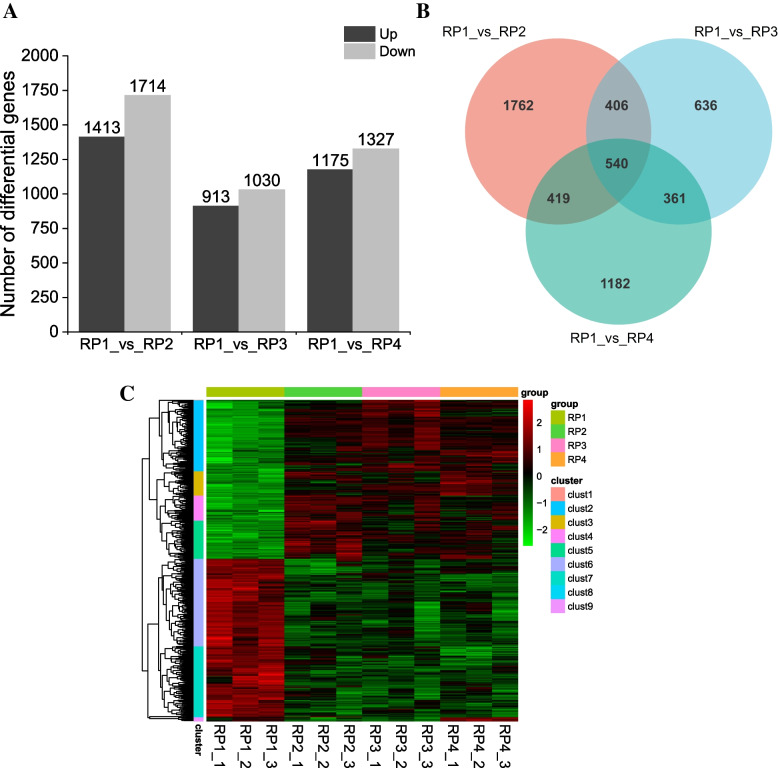
Screening and clustering analysis of differential genes in *L. barbarum* fruits harvested at four different periods. **A** Screening of differentially expressed genes. The *X*-axis represents the three comparison groups and the *Y*-axis represents the number of DEGs. **B** Venn diagram of the number of DEGs in the three comparison groups. **C** Cluster heat map analysis of 540 identical DEGs from three comparison groups

The expression patterns and transcriptional differences of genes in mature *L. barbarum* fruits harvested at four different periods were further analyzed by using the statistical method of FPKM. We obtained 7,572 differentially expressed genes (DEGs) under the screening condition of |log_2_(FoldChange)|> 1 and significance *P*-value < 0.05, using RP1 as the control. It was observed that RP1 had 3,127 DEGs compared with RP2, of which1,413 DEGs were up-regulated and 1,714 DEGs were down-regulated. In the comparison group of RP1 vs RP3, 1,943 DEGs were screened, including 913 up-regulated and 1030 down-regulated genes. RP1 vs RP4 had a total of 2,502 DEGs, among them 1,175 genes were up-regulated and 1,327 genes were down-regulated (Fig. 3A, Fig. S1). Venn diagram shows that the overlap of DEGs between RP1 and RP2, RP3, RP4 comparison groups, and these three comparison groups contained 540 identical DEGs (Fig. 3B). The subsequent clustering analysis revealed that the expression patterns of these 540 DEGs could be clearly distinguished into the control group of RP1 and the treatment groups of RP2, RP3 and RP4 (Fig. 3C). This also indicates that the transcription levels of genes in RP1 are significantly different from those in RP2, RP3, and RP4. Based on the results of clustering heat map, 540 DEGs were divided into 9 clusters according to the similarity of their expression patterns, and the genes in each cluster belonged to one category and might perform similar functions (Fig. S2). These results indicated that the transcription levels of wolfberry fruits harvested at four different periods were extremely different.

### Metabolic pathway analysis of DEGs in *L. barbarum* fruits harvested at four different periods

**Fig. 4:**
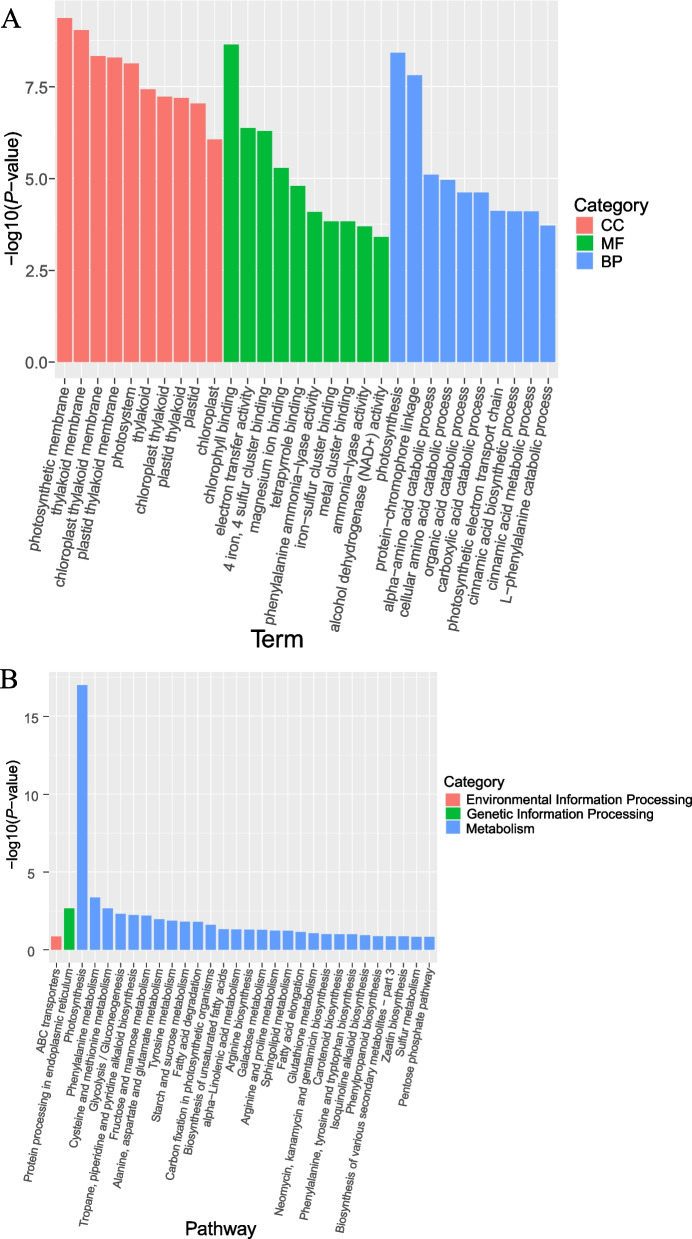
GO enrichment and KEGG metabolic pathway analysis of 540 DEGs. **A** GO functional enrichment analysis of 540 DEGs. The *X*-axis represents GO Term and the *Y*-axis represents -log10(*P*-value), MF: molecular functions, BP: biological processes, CC: cellular components. **B** KEGG pathway enrichment analysis of 540 DEGs. The *X*-axis represents Pathway and the *Y*-axis represents -log10(*P*-value)

To further investigate differentially expressed genes, a total of 540 DEGs in the three comparison groups were then enriched for GO function. There were 341, 132 and 642 terms enriched in cellular component (CC), molecular function (MF) and biological process (BP), respectively. The DEGs were mainly enriched in the photosynthetic membrane and thylakoid membrane in CC. The DEGs were mainly enriched in the chlorophyll binding, electron transfer activity and 4 iron, 4 sulfur cluster binding in MF. The GO terms were significantly enriched by DEGs in BP, including photosynthesis, cellular amino acid catabolic process, organic acid catabolic process, *L*-phenylalanine catabolic process, cinnamic acid biosynthetic and metabolic process (Fig. 4A).

To further understand the metabolic pathways and biological functions involved in DEGs, KEGG metabolic pathway analysis was performed on a total of 540 identical DEGs in the three comparison groups. The KEGG pathway analysis showed that 540 DEGs were enriched to 83 KEGG pathways, among which 15 pathways were significantly enriched. the significantly enriched metabolic pathways included photosynthesis, carbon fixation in photosynthetic organisms, phenylalanine metabolism, cysteine and methionine metabolism, glycolysis / gluconeogenesis, tropane, piperidine and pyridine alkaloid biosynthesis, fructose and mannose metabolism, alanine, aspartate and glutamate metabolism, tyrosine metabolism, starch and sucrose metabolism, fatty acid degradation, biosynthesis of unsaturated fatty acids, alpha-linolenic acid metabolism, and arginine biosynthesis (Fig. 4B). GO and KEGG enrichment analysis showed that these DEGs mainly affected the fruit quality of *L. barbarum* fruits harvested at four periods by regulating the metabolism level of amino acids, sugars, organic acids and secondary substances.

### Cluster heat map analysis of DEGs in lipid, carbohydrate and amino acid metabolism

**Fig. 5:**
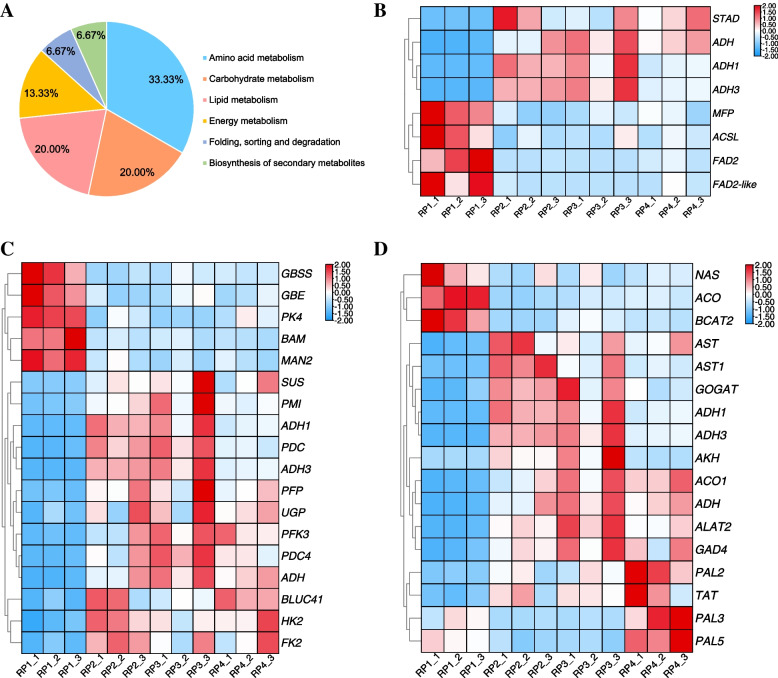
Cluster heat map analysis of DEGs in lipid, carbohydrate and amino acid metabolism. **A** Percentage diagram of 15 metabolic pathways with significant enrichment. **B** Cluster heat map analysis of DEGs involved in lipid metabolism. **C** Cluster heat map analysis of DEGs involved in carbohydrate metabolism. **D** Cluster heat map analysis of DEGs involved in amino acid metabolism. The *X*-axis represents fruit samples of four different periods and the *Y*-axis represents DEGs

Based on the above KEGG enrichment analysis of 540 DEGs, the DEGs involved in amino acid, carbohydrate and lipid metabolism accounted for the highest proportion (Fig. 5A). The DEGs *MFP*, *ACSL*, *FAD2* and *FAD2-like* involved in lipid metabolism pathway showed higher transcription levels in RP1, *ADH*, *ADH1* and *ADH3* were highly expressed in RP2 and RP3, whereas *ADH* showed high transcript levels in RP4. Besides, the *STAD* showed higher transcript levels in RP2 and RP4 than in RP1 and RP3 (Fig. 5B, Table S3). As shown in the graph, most of the DEGs related to carbohydrate metabolism had higher transcript levels in RP2, RP3 and RP4 than in RP1. In contrast, only *GBSS*, *GBE*, *PK4*, *BAM* and *MAN2* genes showed higher transcript levels in RP1 (Fig. 5C, Table S3). The *NAS*, *ACO*, *BCAT2* were expressed at the highest level in RP1, and genes including *AST*, *AST1*, *GOGAT*, *ADH1*, *ADH3*, *AKH* showed high transcript levels in RP2 and RP3; the *ACO1*, *ADH*, *ALAT2*, *GAD4*, *PAL2*, *TAT* genes showed higher transcript levels in RP2, RP3, RP4 than in RP1, and the transcripts of *PAL3* and *PAL5* showed higher levels in RP1 and RP4 than in RP2 and RP3 (Fig. 5D, Table S3). The results of cluster heat map analysis indicated that the transcription levels of DEGs involved in lipid, carbohydrate and amino acid metabolism in RP2 and RP3 were higher than in RP1 and RP4. This means that the abundance of lipids, sugars and amino acids in RP2 and RP3 fruits may be better than RP1 and RP4.

### Separation and identification of metabolites in *L. barbarum* fruits harvested at four different periods

**Fig. 6:**
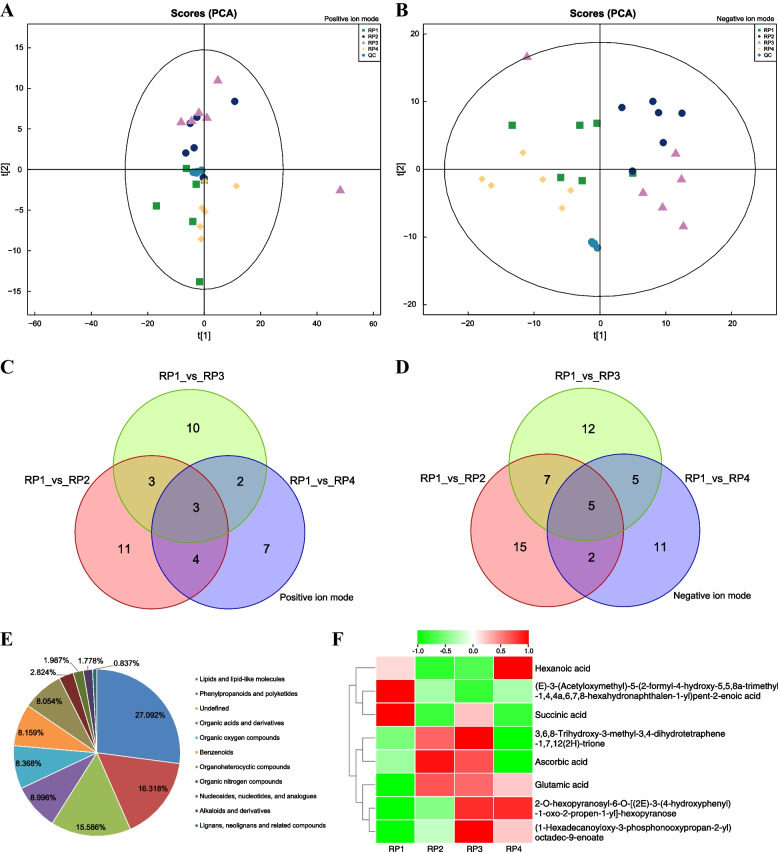
Metabolomic analysis of ripe *L. barbarum* fruits harvested at four different periods. **A** and **B** PCA score plots of metabolite profiles of *L. barbarum* fruits harvested four different periods. t[1] represents the principal component 1, t[2] represents the principal component 2, the ellipse represents 95% confidence interval. Points of the same color indicate each biological repeat within the group, the distribution of points reflects the degree of difference between and within groups. The aggregation degree of QC samples reflects whether repeatability of the experiment is good or bad. **C** and **D** Venn diagram showing numbers of DAMs in three comparison groups. **E** The proportion of metabolites identified in *L. barbarum* fruit in each chemical classification. Different color blocks express different chemical classification items, the percentage represents the number of metabolites in the chemical classification items as a percentage of the total number of identified metabolites. Metabolites without chemical classification are defined as undefined. **F** Cluster heat map analysis of 8 identical DAMs from three comparison groups. **A**, **C** is positive ion mode and **B**, **D** is negative ion mode, QC represents the quality control

Previous studies had focused mainly on the changes in the content of metabolites during the development stage of *L. barbarum*, and there has been no comprehensive analysis of the metabolites of *L. barbarum* fruits ripening in different periods. Therefore, the metabolites of ripe wolfberry fruits harvested at four different periods were profiled using UPLC-MS in this study. From the metabolite determination and analysis, a total of 956 metabolites (including 532 in positive ion mode and 424 in negative ion mode) (Table S4) were identified from the collected Goji berries. The 956 metabolites identified were chemically classified, and 16.318% and 8.996% of the metabolites were classified as phenylpropanoids and polyketides and organic acids and their derivatives, respectively. In addition, 15.586% of the metabolites could not be chemically classified (Fig. 6E).

### Analysis of differentially accumulated metabolites in *L. barbarum* fruits harvested at four different periods

Firstly, quality control (QC) analysis of the samples showed that the correlation coefficient between QC samples was greater than 0.9 and the number of peaks with relative standard deviation RSD ≤ 30% accounted for more than 80% of the total number of peaks in QC samples (Fig. S3), indicating that the instrumental analysis system was stable and the data could be used for subsequent analysis. Then, multivariate statistical analysis by unsupervised PCA and supervised OPLS-DA was performed to support pattern recognition of metabolic differences in Goji fruit samples. In the positive and negative ion mode, the PCA model explained the variation degree of 56.50% and 56.20% between the samples in each group (Table S5). The PCA plots showed that parallel samples of the experimental sample groups RP1s, RP2s, RP3s and RP4s were clustered together, which indicated that the sample selection was reasonable and that there were differences between the metabolites in the sample groups (Fig. 6A, B). Similarly, the OPLS-DA analysis showed clear distinctions among the three groups (Fig. S4). The values of the model evaluation parameters R2Y and Q2 (> 0.5) in the positive ion mode were 0.989 and 0.796 in RP1 vs RP2, 0.958 and 0.841 in RP1 vs RP3, 0.992 and 0.781 in RP1 vs RP4, respectively, and in the negative ion model is similar (Table S5). These results indicated that the OPLS-DA model had high reliability and was suitable for evaluating the differences between the three groups.

Based on the VIP values obtained from the OPLS-DA model and the *P*-value from the univariate analysis, a total of 136 DAMs were screened according to VIP > 1 and *P*-value < 0.05. 50 DAMs (24 up-regulated and 26 down-regulated) were identified between RP1 and RP2; 47 DAMs (26 up-regulated and 21 down-regulated) were identified between RP1 and RP3; 39 DAMs (29 up-regulated and 10 down-regulated) were identified between RP1 and RP4 (Table S4). It could be seen that the accumulation level of DAMs in RP1, RP3 and RP4 was higher than that in RP2. Venn diagram showed that the number of unique differential metabolites is highest for RP1 vs RP2 (26 DAMs), RP1 vs RP3 was the second (22 DAMs), and RP1 vs RP4 was the least (18 DAMs) (Fig. 6C, D). Furthermore, there were 8 shared differential metabolites in the three comparison groups (3 in positive ion mode and 5 in negative ion mode) (Table S6). The (E)-3-(Acetyloxymethyl)-5-(2-formyl-4-hydroxy-5,5,8a-trimethyl-1,4,4a,6,7,8-hexahy dr onaphthalen-1-yl) pent-2-enoic acid and Succinic acid accumulated mainly in RP1; Three compounds including3,6,8-Trihydroxy-3-methyl-3,4-dihydrotetraphene-1,7,12(2H)-trione, Ascorbic acid and Glutamic acid accumulated much more in RP2 and RP3; (1-Hexadecanoyloxy-3-phosphonooxypropan-2-yl) octadec-9-enoate and 2-O-hexopyranosyl-6-O-[(2E)-3-(4-hydroxyphenyl)-1-oxo-2-propen-1-yl]-hexopyranose accumulated at higher levels in RP3 and RP4; Hexanoic acid is mainly accumulated in RP4 (Fig. 6F). The accumulation of these eight differential metabolites in *L. barbarum* fruits at four different periods was consistent with the metabolite relative content plots (Fig. S6). Based on the clustered heat map results, it was found that the accumulation abundance of these eight shared differential metabolites in PR3 fruits was superior to RP1, RP2 and RP4.

### KEGG pathway analysis of DAMs in *L. barbarum* fruits harvested at four different periods

**Fig. 7:**
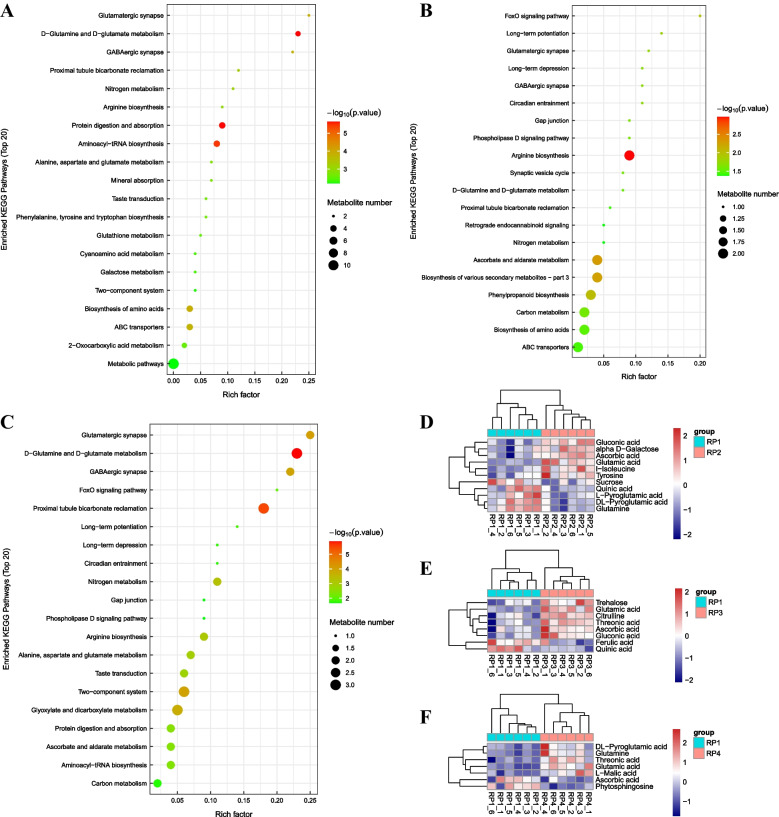
KEGG pathway and cluster heat map analysis of DAMs in the three comparison groups. **A-C** represent the comparison groups RP1 vs RP2, RP1 vs RP3 and RP1 vs RP4, respectively. The bubble color indicates the *P*-value, the darker the bubble color, the smaller the *P*-value, and the more significant the enrichment degree. The bubble size indicates the influence factor size of the path in topology analysis, the larger the bubble, the greater the influence factor. **D-F** represent the RP1 and RP2, RP1 and RP3, RP1 and RP4, respectively. The *X*-axis represents fruit samples and the *Y*-axis represents DAMs. Red represents up-regulated, blue represents down-regulated, color depth indicates the degree of up and down. The red arrow indicates the same differential metabolite ascorbic acid accumulated in the three comparison groups. The green arrow indicates the same differential metabolite glutamic acid accumulated in the three comparison groups

To explore the potential metabolic pathways involved in quality-related DAMs in Goji fruits harvested at four different periods, then submitted all DAMs screened in each comparison group to the KEGG database for metabolic pathway analysis. Three comparison groups have screened that the DAMs annotated 71, 46 and 50 KEGG pathways, among which 37, 21 and 29 KEGG pathways changed significantly (*P*-value < 0.05), respectively. The KEGG pathway involved in the metabolic process in RP1 and RP2 mainly included *D*-glutamine and *D*-glutamate metabolism, alanine, aspartate and glutamate metabolism, glutathione metabolism, galactose metabolism, arginine biosynthesis, phenylalanine, tyrosine and tryptophan biosynthesis (Fig. 7A); In RP1 and RP3, the DAMs were mainly involved in metabolic processes of *D*-glutamine and *D*-glutamate metabolism, biosynthesis of amino acids, arginine biosynthesis, ascorbate and aldarate metabolism, biosynthesis of various secondary metabolites, phenylpropanoid biosynthesis (Fig. 7B); Similarly, the differential metabolites in RP1 and RP4 were involved in *D*-glutamine and *D*-glutamate metabolism, arginine biosynthesis, alanine, aspartate and glutamate metabolism, ascorbate and aldarate metabolism (Fig. 7C). It was observed that *D*-glutamine and *D*-glutamate metabolism were significant enrichment pathways in all three comparison groups. These results indicate that the DAMs in the three comparison groups were mainly enriched in amino acid biosynthesis and metabolism and carbohydrate metabolism, which was similar to the KEGG enrichment results of the transcriptome.

To facilitate the observation of the expression of each differential metabolite annotated in the KEGG metabolic pathway, the KEGG metabolic pathway with the number of differential metabolites more than 5 was selected and the results showed that compared with RP1, the contents of AsA and glutamic acid (Glu) were significantly different in all three comparison groups. In agreement with our previous results, the Vc content was higher in RP2 and RP3 than in RP1, and lowest in RP4. In contrast, the glutamate content was significantly higher in RP2 and RP3 and RP4 than in RP1. In addition, the cluster heat map results showed that the abundance and accumulation of metabolites in RP2, RP3 and RP4 were higher than RP1, which also indicated that the fruit quality of RP1 was not the best (Fig. 7D-F).

### Integrative analysis of transcriptome and metabolome to explore the quality differences of* L. barbarum* fruits harvested at four different periods

In order to identify potential quality-related pathways between four different periods of harvesting, we identified DAMs based on the metabolome and then searched for differential transcripts associated with them to obtain a common KEGG pathway for DAMs and DEGs. The transcriptome and metabolome association analysis showed that the differential genes involved in phenylpropane biosynthesis, ascorbic acid and succinic acid metabolism accounted for a relatively high proportion (Table S7). Then, relevant genes and metabolites involved in these pathways were extracted to draw the metabolic pathways and expression heat maps.

**Fig. 8:**
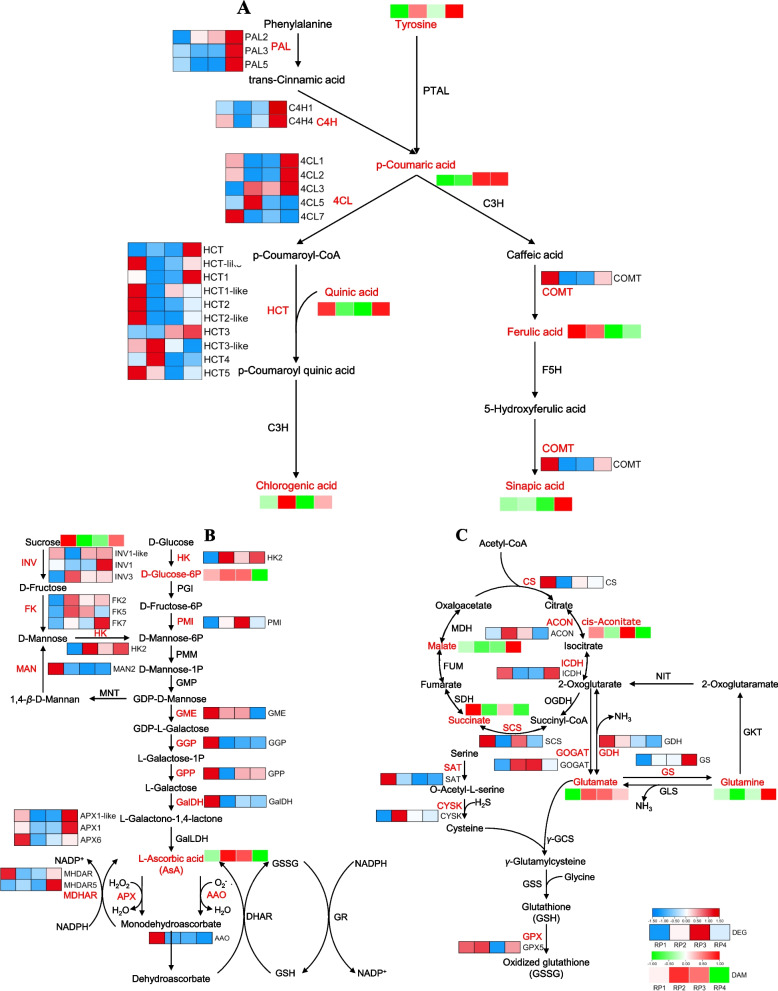
Expression heat map of related genes and metabolites in phenylpropane biosynthesis, ascorbic acid, succinic acid and glutamic acid metabolic pathway. **A** Expression heat map of related genes and metabolites in chlorogenic acid, ferulic acid and sinapic acid biosynthesis pathway. PAL: phenylalanine ammonialyase; C4H: cinnamic acid 4-hydroxylase; 4CL: 4-coumaric acid–CoA ligase; HCT: quinate hydroxycinnamoyl transferase; C3H: *ρ*-coumaroylester 3′-hydroxylase; PTAL: phenylalanine/tyrosine ammonia-lyase; F5H: ferulate-5-hydroxylase; COMT: caffeic acid 3-O-methyltransferase. **B** Expression heat map of related genes and metabolites in ascorbic acid metabolic pathway. INV: beta-fructofuranosidase; FK: fructokinase; HK: hexokinase MAN: mannan endo-1,4-beta-mannosidase; MNT: mannan 1,4-beta-D-mannosyltransferase; PGI: glucose-6-phosphate isomerase; PMI: mannose-6-phosphate isomerase; PMM: phosphomannomutase; GMP: GDP-mannose pyrophosphorylase; GME: GDP-mannose-3',5'-epimerase; GGP: GDP-*L*-galactose phosphorylase; GPP: *L*-galactose 1-phosphate phosphatase; GalDH: *L*-galactose dehydrogenase; AAO: *L*-ascorbate oxidase; APX: *L*-ascorbate peroxidase; DHAR: dehydroascorbate reductase; MDHAR: monodehydroascorbate reductase; GSH: Reduced glutathione; GSSG: Oxidized glutathione; GR: glutathione reductase; **C** Expression heat map of related genes and metabolites in succinic acid and glutamic acid metabolic pathway. CS: citrate synthetase; ACON: aconitate hydratase; ICDH: isocitrate dehydrogenase; OGDH: 2-oxoglutarate dehydrogenase; SCS: succinyl-CoA synthetase; SDH: succinate dehydrogenase; FUM: fumarase; MDH: malate dehydrogenase; NIT: 2-oxoglutaramate amidase; GKT: glutamine-ketoacids transaminase; GOGAT: glutamate synthetase; GDH: glutamate dehydrogenase; GS: glutamine synthetase; GLS: glutaminase; SAT: serine acetyltransferase; CYSK: cysteine synthase; *γ*-GCS: gamma-glutamylcysteine synthetase; GSS: glutathione synthetase; GPX: glutathione peroxidase

There were 21 single genes encoding 5 putative enzymes phenylpropane biosynthesis pathway, including phenylalanine ammonialyase (PAL, EC: 4.3.1.24), cinnamic acid 4-hydroxylase (C4H, EC: 1.14.14.91), 4-coumaric acid–CoA ligase (4CL, EC: 6.2.1.12), quinate hydroxycinnamoyl transferase (HCT, EC: 2.3.1.133) and Caffeic acid 3-O-methyltransferase (COMT, EC: 2.1.1.68) (Table S8). These enzymes were identified to be involved in the biosynthesis of coumaric acid, chlorogenic acid, ferulic acid and sinapic acid. In addition, it has been found that coumaric acid was involved in the enzymatic reaction substrate of these two pathways, three *PAL* and two *C4H* genes were up-regulated in RP4, and the metabolite metabolite *p*-coumaric acid was significantly accumulated in RP3 and RP4. The transcription levels of *4CL3* in RP2, RP3 and RP4 were higher than that in RP1. Three *HCT* (*HCT*, *HCT1*, *HCT3*) genes were up-regulated in RP4. Meanwhile, *HCT3-like* and *HCT4* genes were up-regulated in RP2, and one *COMT* gene was up-regulated in RP1. The corresponding metabolites chlorogenic acid were mainly accumulated in RP2 and RP4, ferulic acid accumulates more in RP1 and RP2, and sinapic acid was significantly accumulated in RP4 (Fig. 8A).

There were 12 single genes encoding 8 putative enzymes in the AsA metabolic pathway, including hexokinase (HK, EC: 2.7.1.1), mannose-6-phosphate isomerase (PMI, EC: 5.3.1.8), and GDP-mannose-3',5'-epimerase (GME, EC: 5.1.3.18), GDP-*L*-galactose phosphorylase (GGP, EC: 2.7.7.69),* L*-galactose 1-phosphate phosphatase (GPP, EC: 3.1.3.93), *L*-galactose dehydrogenase (GalDH, EC: 1.1. 1.316), *L*-ascorbate oxidase (AAO, EC: 1.10.3.3), *L*-ascorbate peroxidase (APX, EC: 1.11.1.11) and monodehydroascorbate reductase (MDHAR, EC: 1.6.5.4). Usually, sucrose metabolism could provide intermediate metabolites for AsA biosynthesis, our results indicated that in the AsA metabolic pathway the upstream *GME* showed low transcript levels in RP4, while the downstream gene *MHDAR5* showed high transcript levels in RP4. In RP1, the upstream genes *GGP*, *GalDH* and downstream gene *AAO* all showed high transcript levels. However, the transcript level of upstream genes *GPP* showed low transcript levels in RP2. In addition, upstream gene *PMI* showed higher transcript levels in RP2 and RP3 than in RP1 and RP4. Interestingly, downstream genes including *APX1-like*, *APX1*, *APX6* and *MHDAR* showed higher transcript levels in RP1 and RP4 than in RP2 and RP3, and the expression patterns of these genes were consistent with the trend of AsA efficient accumulation (Fig. 8B).

In general, the metabolites succinate and glutamate identified in all three comparison groups could form the following network metabolic map (Fig. 8C). The expression pattern of the *SCS*, encoding succinyl-CoA synthetase (SCS, EC: 6.2.1.5), was consistent with the trend of succinate accumulation in fruit at all four periods, which significantly accumulated in both RP1 and RP3. Similarly, the expression pattern of the gene *GOGAT* encoding the glutamate synthase (EC: 1.4.1.14) followed the same trend as the accumulation pattern of Glu, which performed higher transcript levels in RP2, RP3 and RP4 than in RP1, and the accumulation of Glu was also significantly higher in RP2, RP3 and RP4 than in RP1. Because of the Glu and glutamine can be interconverted under certain conditions, which involved in the glutamine synthetase (GS, EC: 6.3.1.2), our results found that the transcript level of *GS* was highest in RP4 and the highest glutamine content was also appeared in RP4 (Fig. 8C). Nevertheless, Glu and glutamine is one of the key substances in reduced glutathione biosynthesis, and we found that AsA and Glu were important substances in the ascorbate–glutathione recycling system (AsA-GSH). AsA-GSH is an important component of the non-enzymatic antioxidant system, which can effectively scavenge reactive oxygen species (ROS) produced in plants and maintain redox balance.

### Verification of DEGs by qRT‑PCR and enzyme activity analysis

**Fig. 9:**
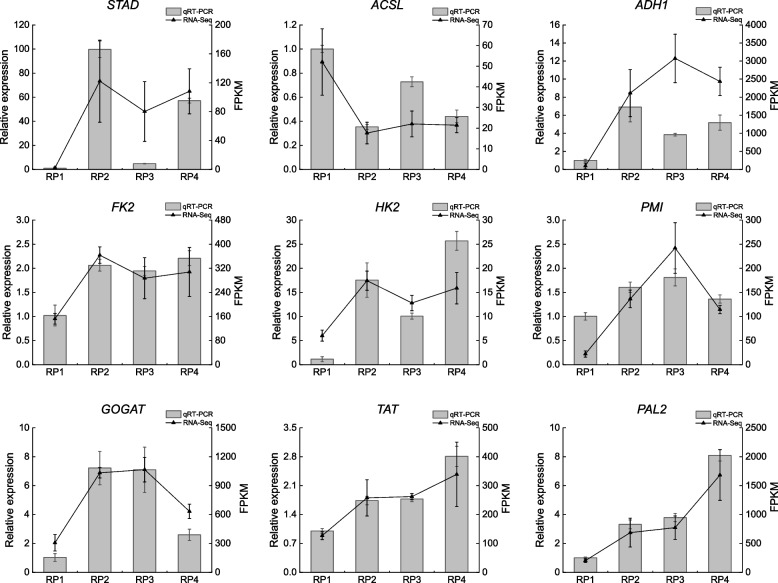
Transcriptional levels of genes related to lipid, carbohydrate and amino acid metabolism were analyzed by qRT-PCR assay

In order to verify the reliability of the transcriptome sequencing experiments and the accuracy of the sequencing data, nine DEGs involved in lipid, carbohydrate, and amino acid metabolism *STAD* (*Lba10g00375*), *ACSL* (*Lba06g01326*), *ADH1* (*Lba01g01582*), *FK2* (*Lba01g02501*), *HK2* (*Lba01g02006*), *PMI* (*Lba12g00413*), *GOGAT* (Lba03g01605), *TAT* (Lba01g00166) and *PAL2* (*Lba08g01407*) were subjected to expression validation analyses by qRT-PCR. The results showed that the expression trends of the eight DEGs except *ADH1* in the wolfberry fruits at the four periods were basically consistent with the transcriptome sequencing results, which indicated that the results of RNA-Seq analysis were reliable (Fig. 9).

**Fig. 10:**
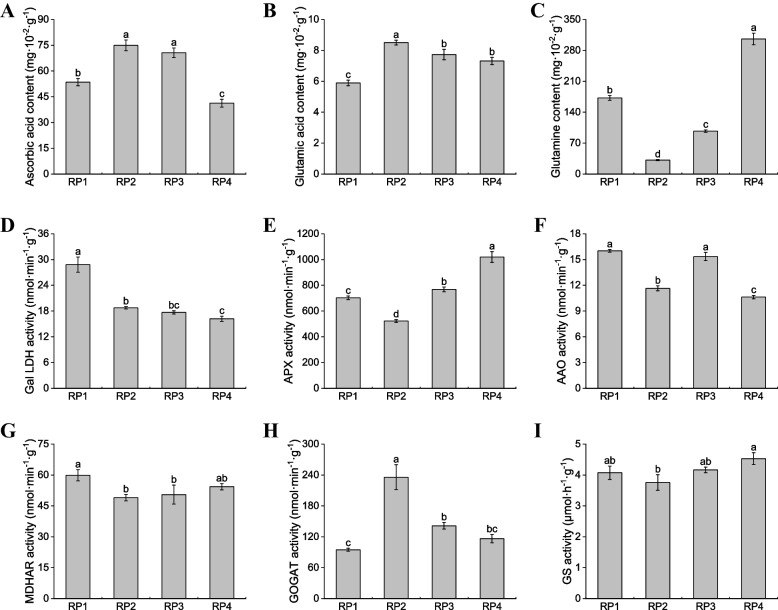
Analysis of critical metabolites and enzyme activities in the ascorbic acid and glutamate biosynthetic pathways. **A-C** Analysis of differential metabolite accumulation patterns in *L. barbarum* fruits harvested at four different periods. **D-I** Analysis of GalLDH, APX, AAO, MDHAR, GOGAT, and GS activities in *L. barbarum* fruits harvested at four different periods. Each column shows the average value of three replicated experiments, and bars indicate the standard error of the value. Different lowercase letters denote significant differences at *P* < 0.05

Through transcriptome and metabolome association analysis, it was found that metabolome identified metabolites ascorbic acid and glutamic acid as important substances in AsA-GSH. The contents of AsA, Glu and glutamine (Gln) as well as the related enzyme activities were further detected and analyzed in *L. barbarum* fruits harvested at four different periods. The results showed that the accumulation patterns of AsA, Glu and Gln in wolfberry fruits were similar to those of metabolomes (Fig. 10A-C). Through the analysis of related enzyme activities, it was found that the GalLDH activity was the highest in RP1, followed by RP2, and the APX, AAO and MDHAR activities were relatively low in RP2 (Fig. 10D-G). This suggests that high activity GalLDH and low activity APX, AAO can promote AsA accumulation in ripe fruits. The activity of GOGAT in RP2 was significantly higher than that in other periods, while the activity of GS in RP4 was higher (Fig. 10H, I). Meanwhile, the accumulation of Glu in RP2 and Gln in RP4 was the highest, both of which were significantly higher than other periods (Fig. 10B, C). This suggests that the high activity of GOGAT and GS in ripe wolfberry fruits is necessary for the synthesis of more Glu and Gln, there by improving the nutritional value of the fruits.

## Discussion

The importance of berry fruits in a healthy and balanced diet is increasingly valued by dietitians and health care professionals because they contain a variety of rich and effective phytochemical [39]. *L. barbarum* is a shrub that can blossom and bear fruit continuously from May to September, and the berries can ripen successively [21]. Wolfberry fruit is popular in the consumer market as a food rich in nutrients and health benefits [4]. In *L. barbarum* plants, both the fruit ripening time and fruit quality regulation is not only a basic research topic, but also a significant agronomic trait [12]. However, compared with the study of fruits from other fruit trees, the study of ripening and quality regulation in wolfberry fruits remains scarce. Therefore, studying the quality difference of wolfberry fruits harvested at different periods can help improve the quality of berries during the harvest season.

Berry size is highly important in Goji berry cultivation and fresh consumption. The length, width, and weight of wolfberry fruits. The length, width, and weight of wolfberry fruits reached the maximum at the beginning of the harvest season, but showed a decreasing trend in the subsequent picking of the harvest season [21]. The largest weight of berries was harvested in June, while the smallest weight of berries was picked in September [20, 21]. The results of this study also showed that berries picked at the beginning of the harvest season had the highest 100-grain fresh weight for RP1 (69.34 g), while berries picked at the end of the harvest season had the lowest 100-grain fresh weight for RP4 (56.48 g). In addition, *L. barbarum* fruits (RP1, RP2 and RP3) harvested in summer were higher in 100-grain fresh weight, 100-grain dry weight and fresh weight/dry weight than the berries (RP4) harvested in autumn (Table 1). This may be due to a lower crop load at the beginning of the harvest season, so fruit size and weight are relatively high.

The content of total soluble solids (TSS) is an important index for evaluating the quality and freshness of wolfberry. The TSS of wolfberry is mainly composed of sugar, acid, pectin, vitamins and other substances [40]. The TSS content was lowest in the first fruiting month (June) and then increased steadily, reaching the maximum in the last month (September) [21]. The TSS content of wolfberry fruits picked in July and August was higher than that of fruits picked in June and September [20]. The results of this study showed that the highest TSS content was 17.88% in berries picked on July 19 (RP3), followed by September 20 (RP4, 17.05%). The TSS content of the berries RP1 and RP2 harvested at the beginning of the production season was around 15% (Fig. 1B). At the beginning of the harvest season, fruit growth will be faster because the crop load is low, the fruiting branches are full of nutrients and the phytohormone metabolism is high [21]. However, this rapid growth and development gives the fruit a larger intercellular space and leads to a reduction in the composition of dry matter per unit volume [41]. Goji berry is one of the natural sources of carotenoids, and the average carotenoid content in red wolfberry is 233.04 mg∙10^–2^∙g^−1^ [14]. Carotenoids are not only an important factor in determining the appearance quality of wolfberry fruit, but also an important indicator of the intrinsic quality of wolfberry fruit [42]. The carotenoid content in the fruit was determined at different harvesting periods and found to be higher in summer fruit than in autumn fruit [19]. The carotenoid contents of our June and July harvested berries RP1, RP2 and RP3 were 31.55, 31.51 and 31.75 mg∙10^–2^∙g^−1^, respectively, which were higher than those of the September harvested berries RP4 (29.08 mg∙10^–2^∙g^−1^) (Fig. 1G). Therefore, our results also indicated that the carotenoid content of *L. barbarum* fruits harvested in summer was higher than that of fruit harvested in autumn. The carotenoid content of *L. barbarum* fruits harvested in summer were better.

Usually, fruit sweetness is mainly determined by soluble sugars, acidity is mainly controlled by organic acids, and the ratio of sugar-to-acid in fruit determines the flavor to some extent [43]. The total sugar in ripe fruits is mainly composed of three components: fructose, glucose and sucrose [24]. Glucose is the main sugar in wolfberry fruits, followed by fructose and sucrose [18]. Fructose and glucose content continuously increase during fruit development, while sucrose content with ripening and accumulates mainly in the form of glucose and fructose at maturity [23]. The total sugar content of ripe Goji berries ranged from 10.1% ~ 13.8% [18]. The total sugar content in the fruits of *L. barbarum* collected at four different periods ranged from 8.76% ~ 10.29%, which was similar to the results of Oğuzn et al. [18]. Zorenc et al. [44] reported that the monosaccharide and total sugar content in berry fruits generally increased during successive harvests, with the highest total sugar content in berry fruit picked on the last harvest date. Our Goji berries RP1 picked on June 17 had the highest total sugar content of 10.29%, followed by RP4 (10.19%). In addition, the highest reducing sugar content in RP1 fruits was 9.80%. Due to the low load on the *L. barbarum* plants at the beginning of the harvest season, the fruiting branches are well-nourished. The assimilation products of the plant are distributed to fewer fruits, allowing for better fruit sugar accumulation. During the ripening period of *L. barbarum* fruit, the content of LBPs ranged from 13.03 to 76.86 mg∙g^−1^ (FW), the ‘Ningji 1’ ripe fruits LBPs content is as high as 48.6 mg∙g^−1^ [23, 45]. We obtained the highest polysaccharide content of 2.13% in the Goji berries at the last harvest, followed by RP3 and RP1 with 1.67% and 1.56%, respectively. RP1 performed relatively well in sugar content among *L. barbarum* fruits harvested at four different periods. The following RNA-seq and KEGG analysis of Goji berry fruits showed that a total of 540 DEGs from the three comparison groups were enriched to three pathways closely related to sugar metabolism. The genes of the pathways related to glucose metabolism were screened mainly included 18 genes such as *SUS* (sucrose synthase), *UGP* (UTP–glucose-1-phosphate uridylyltransferase), *HK2*, *FK2*, *PFK* (6-phosphofructokinase), *PMI* etc. These genes were differentially expressed in the fruits of four different periods, and most of their DEGs had higher transcript levels in RP2, RP3, RP4 than RP1 but genes including *CBSS*, *GBE*, *PK4*, *BAM*, and *MAN2* had higher transcript levels in RP1.

Organic acids in food and beverages enhance sensory characteristics by providing flavor, color, and aroma, and different fruits contain different types and compositions of organic acids that give the fruit its unique flavor [46]. Citric acid, tartaric acid and quinic acid are the main organic acid components in the developing fruit of *L. barbarum*, and their contents showed a trend of increasing and then decreasing with fruit growth and development, where the contents of citric acid, tartaric acid and quinic acid ranged from 12.24 to 17.62 mg∙g^−1^ (fresh weight, FW), 1.14 to 4.66 mg∙g^−1^ (FW) and 0.80 to 4.07 mg∙g^−1^ (FW), respectively [23]. Malic and succinic acid contents in ‘Ningqi 1’ were 0.08 and 0.58 mg∙g^−1^ (FW), respectively [28]. We also identified organic acids such as quinic acid, succinic acid, and malic acid (*L*-Malic acid) in Goji fruits harvested at four different periods by untargeted metabolomics techniques. The accumulation of these three organic acids varied among the four periods, with quinic acid accumulating mainly in RP1 and RP4, succinic acid in RP1 and RP3, and malic acid in RP4. The expression pattern of *SCS* gene in the succinic acid metabolic pathway was consistent with the trend of succinic acid accumulation in the fruit. Besides, fruit quality is also influenced by amino acid accumulation [47]. It has been shown that alanine, threonine, and serine are associated with sweetness in grapes, while glutamic acid and aspartic acid are associated with sourness [48], and asparagine and alanine affect the flavor of soybeans [49]. We detected the accumulation of glutamate in the fruit at all four periods. In addition, transcriptomic analysis showed that the expression pattern of *GOGAT*, a glutamate synthase gene in the alanine, aspartate, and glutamate metabolic pathways, was similar to the accumulation pattern of glutamate in the fruits of the four periods. In addition, glutamate and glutamine can be interconverted under certain conditions, our study indicated that the glutamine synthase gene *GS* having the highest transcript level in RP4 and the corresponding is the highest glutamine content in RP4.

Notably, Goji berry is rich in ascorbic acid, thiamin, riboflavin and vitamins E, B1, B2 and B6 [18, 50]. Compared to the other five *Lycium* varieties in ‘Ningji 1’ the ascorbic acid content was up to 55.05 mg∙10^–2^∙g^−1^ in ripening fruit [28]. We determined the ascorbic acid content in four different periods of harvesting, and the ascorbic acid content was highest in RP2 (74.94 mg∙10^–2^∙g^−1^) followed by RP3 (70.66 mg∙10^–2^∙g^−1^), and significantly lower in RP1 than in RP2 and RP3, but higher than in RP4, which was consistent with our metabolomic results in the accumulation mode. The biosynthetic pathways of AsA differ in different organs or different growth and developmental stages in plants, and four major synthetic pathways have been proposed, among which the *D*-mannose/*L*-galactose pathway is recognized as the major pathway for AsA synthesis in plants [51]. This study suggests that the main pathway for AsA synthesis in *L*. *barbarum* fruit may be the *L*-galactose pathway. GME is considered to be the central enzyme of AsA biosynthesis and a key regulator of AsA accumulation and cell wall biosynthesis [52]. GGP, a key rate-limiting enzyme in the synthesis of *L*-galactose, catalyzes the production of GDP-*L*-galactose-1-phosphate [53]. The key rate limiting enzyme gene *GGP* of *L*-galactose pathway showed high expression level in RP1, expression of upstream genes *HK* and *GME* was the lowest in RP1 and RP4, respectively, downstream genes *GalDH* and *AAO* showed high expression levels in RP1, *MHDAR5* gene showed the highest transcription level in RP4, and *GPP* showed low expression level in RP2. In addition, the upstream gene *PMI* showed higher transcript levels in RP2 and RP3 than in RP1 and RP4, and conversely, downstream genes *APX1-like*, *APX1*, *APX6* and *MHDAR* genes showed higher transcript levels in RP1 and RP4 than in RP2 and RP3, and the expression patterns of these genes were similar to the trend of AsA accumulation. Among the tomato *GME* family, *SlGME1* is expressed in different tissues, whereas *SlGME2* is expressed differently in different tissues. Tomato transgenic plants overexpressing *SLGME1* and *SLGME2* showed enhanced stress tolerance and increased total AsA content in both leaves and fruits [54]. Overexpression of kiwifruit fruit *GGP* genes in *Arabidopsis* led to a fourfold increase in AsA content, while transient overexpression of *GGP* and *GME* genes in tobacco leaves resulted in a sevenfold increase [53, 55].

With the rapid development of bioinformatics, more and more scholars are using the transcriptome, proteome and metabolome to provide a broad perspective for the study of fruit quality traits and metabolic basis [56]. By sequencing the transcriptome of ‘Ningqi No.1’ Goji berries, it was found that *GAE*, *GALA* and *MS* play a key role in the regulation sugar metabolism in *L. barbarum* fruits under elevated CO_2_ conditions [57]. RNA sequencing of *L. chinense* wolfberry using a sequencing platform showed that most genes in *L. chinense* were related to phenylpropane biosynthesis, among which *LcPAL*, *LcC4H*, *LcF3'H*, *Lc3GT*, *LcC3H*, *LcCOMT*, *LcCHS*, *LcCHI*, *LcF3H*, and *LcFLS* were highly expressed in ripe fruits, and *Lc4CL* was highly expressed in roots [58]. In this study, RNA-Seq and KEGG analyses were performed on Goji berries harvested at four different periods. The results showed that 18 DEGs were involved in the glucose metabolism pathway, among which most of the DEGs related to glucose metabolism were transcribed at higher levels in RP2, RP3, and RP4 than in RP1, and only the *GBSS*, *GBE*, *PK4*, *BAM*, and *MAN2* genes showed high levels of expression in RP1. The high level of expression of these genes in mature fruits may contribute to the biosynthesis and accumulation of sugars in wolfberry fruits. Studies of Goji berries at five developmental stages using transcriptomics and metabolomics revealed that *AAT1*, *metE*, *pip*, and rutin, raffinose, galactinol, trehalose, citrulline and *DL*-arginine may be of interest to future functional studies of stress adaptation in plants [27]. In this study, we found that *PAL*, *C4H*, *4CL*, *PMI*, *APX*, *MHDAR*, *GOGAT*, *GS*, and chlorogenic acid, ferulic acid, ascorbic acid, glutamic acid, and glutamine were important in regulating the fruit quality at different harvesting periods using transcriptomic and metabolomic studies on *L. barbarum* fruits harvested at four different periods.

The most important mechanism for AsA regeneration in plant cells is the AsA-GSH cycle system. Components such as ascorbic acid, glutamate, glutamine and their related genes play a crucial role in the AsA-GSH pathway and are considered to be key players in H_2_O_2_ metabolism and the AsA cycle [59]. After synthesized in plants, AsA is used as an electron donor to reduce H_2_O_2_ or O_2_^−^_·_ to H_2_O by APX or AAO. While scavenging ROS, AsA is oxidized to produce monodehydroascorbic acid (MDHA). A portion of MDHA is re-reduced to be AsA by MDHAR, which uses NADPH as the reductant, and the other MDHA undergoes a non-enzymatic disproportionation reaction to produce dehydroascorbic acid (DHA) [60]. GSH is oxidized to form GSSH, and GSSH can be reduced again to GSH by glutathione reductase (GR) using NADPH as a cofactor [59]. AsA regeneration requires a continuous supply of GSH and NADPH, and the pathway that supplies these reductant molecules lies outside the AsA biosynthesis mechanism [61]. Thus, after the AsA-GSH cycle ROS are finally scavenged and the AsA-GSH cycle is a key process for the reductive regeneration of ASA in the organism.

## Conclusion

In this study, we explored the regulatory network at the transcriptional and metabolic level for the differences in fruit quality of *L. barbarum* harvested at different periods. The content of vitamin C and carotenoids in wolfberry fruits picked in summer are higher than that in autumn fruits, and has better appearance quality and commodity value, which is suitable for making traditional herbal medicine or health food. The accumulation of sugar substances in wolfberry fruits picked in autumn is relatively rich, which is suitable for making fruit juice, fruit vinegar or fruit wine. The *L. barbarum* fruits harvested in summer have high nutritional and medicinal value, as well as good economic benefits, which is more favored by consumers and growers. In addition, we found that phenolic acids, ascorbic acid and glutamic acid are mainly accumulated in the wolfberry fruits harvested in summer, and their biosynthesis was closely regulated by the expression of related genes. Ascorbic acid and glutamic acid are important players in the ascorbate–glutathione (AsA-GSH) recycling system. Ascorbic acid, phenolic substances and the AsA-GSH recycling system have antioxidant effects, which makes the summer harvest of Goji berries more in line with market demand and health care concepts. Our research results provide a theoretical basis for the rational use of agricultural techniques to improve the nutritional quality and medicinal value of *L. barbarum* fruits harvested at different periods.

### Supplementary Information

**Additional file 1:** **Figure S1.** Volcano plot showing the number of up-regulated or down-regulated different expression genes in different comparison groups. The *X*-axis represents log2(Fold Change) and the *Y*-axis represents -log10(*P*-value). The two vertical dashed lines represent the threshold of expressing the multiple of difference and the horizontal dashed lines represent the threshold of significance level. Different colors of red, blue and white represent up-regulated, down-regulated and no significant difference of DEGs, respectively. **Figure S2.** 540 DEGs expression trend analysis. The *X*-axis represents four samples of fruit from different periods and the *Y*-axis represents log2(fpkm+1). **Figure S3.** Quality control (QC) analysis of samples. (A) and (B) QC sample correlation map. The points in each small square represent the ion peaks (metabolites) extracted from QC samples. The* X*-axis and* Y*-axis represent the logarithm of the ion peak signal intensity value. (C) and (D) Relative standard deviation diagram of QC samples. (A), (B) is positive and (C), (D) negative ion mode. **Figure S4.** OPLS-DA score plots of metabolite profiles of* L. barbarum *fruits. t[1] represents the principal component 1, t[2] represents the principal component 2, the ellipse represents 95% confidence interval. Points of the same color indicate each biological repeat within the group, the distribution of points reflects the degree of difference between and within groups. (A), (C), (E) is positive and (B), (D), (F) negative ion mode, (A), (B) represents the RP1 vs RP2, (C), (D) represents the RP1 vs RP3, (E), (F) represents the RP1 vs RP4. **Figure S5.** Permutation tests of *L. barbarum* fruit samples. The abscissa in the figure represents the degree of substitution retention, and the ordinate represents the values of R2 and Q2. The green point represents R2, the blue point represents Q2, and the two dashed lines represent the regression lines for R2 and Q2, respectively. (A), (C), (E) is positive and (B), (D), (F) negative ion mode, (A), (B) represents the RP1 vs RP2, (C), (D) represents the RP1 vs RP3, (E), (F) represents the RP1 vs RP4. **Figure S6.** The relative contents of 8 identical differentially accumulated metabolites in the three comparison groups obtained by metabolome analysis. The *X*-axis represents *L. barbarum* fruit samples and the *Y*-axis represents relative content. Metabolomic analysis is the relative peak area representing the relative content of the detected substance and the values shown are the means ± SD.

** Additional file 2:** **Table S1.** List of primers. **Table S2.** Mapping features of RNA-seq clean data. **Table S3.** Differentially expressed genes involved in lipid, carbohydrate and amino acid metabolism pathway. **Table S4.** The quantity statistics of differentially accumulated metabolites in *L. barbarum* fruit. **Table S5.** Model evaluation parameters for metabolomics of *L. barbarum* fruit samples. **Table S6.** There are 8 identical differentially accumulated metabolites in the three comparison groups. **Table S7.** Differential expression results of metabolites and related transcripts. **Table S8.** Information on key genes and metabolites related to phenylpropane biosynthesis, ascorbic acid, succinate and glutamate metabolism.

## Data Availability

All data generated and analyzed during this study are included in this published article and its supplementary information files. The raw RNA-seq data generated in the study is available at the SRA database in National Center for Biotechnology Information (NCBI) with the accession number PRJNA936275 (https://www.ncbi.nlm.nih.gov/bioproject/PRJNA936275).

## Abbreviations

AsA: Ascorbic acid
Glu: Glutamic acid
AsA-GSH: Ascorbate-glutathione recycling system
ROS: Reactive oxygen species
LBP: *Lycium barbarum* Polysaccharide
RP1: First ripening period
RP2: Second ripening period
RP3: Third ripening period
RP4: Fourth ripening period
TSS: Total soluble solid
RIN: RNA integrity number
NGS: Next-generation sequencing
FPKM: Fragments Per Kilo bases per Million fragments
DEGs: Differentially expressed genes
GO: Gene Ontology
KEGG: Kyoto Encyclopedia of Genes and Genomes
PCA: Principal Component Analysis
OPLS-DA: Orthogonal Partial Least Squares Discrimination Analysis
VIP: Variable importance for the projection
DAMs: Differential accumulation metabolites
