# Supplementary material for: Integrated metabolome and transcriptome analysis of differences in quality of ripe Lycium barbarum L. fruits harvested at different periods
Source: BMC Plant Biol. 2024 Feb 2;24:82. doi: 10.1186/s12870-024-04751-z (PMC10835843; doi:10.1186/s12870-024-04751-z)
Supplement: Supplementary file 1 — Additional file 1: Figure S1. Volcano plot showing the number of up-regulated or down-regulated different expression genes in different comparison groups. The X-axis represents log2(Fold Change) and the Y-axis represents -log10(P-value). The two vertical dashed lines represent the threshold of expressing the multiple of difference and the horizontal dashed lines represent the threshold of significance level. Different colors of red, blue and white represent up-regulated, down-regulated and no significant difference of DEGs, respectively. Figure S2. 540 DEGs expression trend analysis. The X-axis represents four samples of fruit from different periods and the Y-axis represents log2(fpkm+1). Figure S3. Quality control (QC) analysis of samples. (A) and (B) QC sample correlation map. The points in each small square represent the ion peaks (metabolites) extracted from QC samples. The X-axis and Y-axis represent the logarithm of the ion peak signal intensity value. (C) and (D) Relative standard deviation diagram of QC samples. (A), (B) is positive and (C), (D) negative ion mode. Figure S4. OPLS-DA score plots of metabolite profiles of L. barbarum fruits. t[1] represents the principal component 1, t[2] represents the principal component 2, the ellipse represents 95% confidence interval. Points of the same color indicate each biological repeat within the group, the distribution of points reflects the degree of difference between and within groups. (A), (C), (E) is positive and (B), (D), (F) negative ion mode, (A), (B) represents the RP1 vs RP2, (C), (D) represents the RP1 vs RP3, (E), (F) represents the RP1 vs RP4. Figure S5. Permutation tests of L. barbarum fruit samples. The abscissa in the figure represents the degree of substitution retention, and the ordinate represents the values of R2 and Q2. The green point represents R2, the blue point represents Q2, and the two dashed lines represent the regression lines for R2 and Q2, respectively. (A), (C), (E) is positive and (B [file 12870_2024_4751_MOESM1_ESM.docx]

***BMC Plant Biology* Supplementary Information**

**Integrated metabolome and transcriptome analysis of differences in quality of ripe *Lycium barbarum* L. fruits harvested at different periods**

Deshuai Liu^1, 3^, Miao Yuan^1^, Ye Wang^1, 3^, Li Zhang^1^, Wenkong Yao^1, 2, 3 *^and Mei Feng^1, 2, 3 *^

^1^ College of Enology and Horticulture, Ningxia University, Yinchuan 750021, Ningxia, China

^2^ Ningxia Modern Facility Horticulture Engineering Technology Research Center, Yinchuan 750021, Ningxia, China

^3^ Ningxia Key Laboratory of Modern Molecular Breeding of Dominant and Characteristic Crops, Yinchuan 750021, Ningxia, China

* Correspondence: yaowenkong@163.com and feng_m@nxu.edu.cn





**Figure S1.** Volcano plot showing the number of up-regulated or down-regulated different expression genes in different comparison groups. The *X*-axis represents log2(Fold Change) and the *Y*-axis represents -log10(*P*-value). The two vertical dashed lines represent the threshold of expressing the multiple of difference and the horizontal dashed lines represent the threshold of significance level. Different colors of red, blue and white represent up-regulated, down-regulated and no significant difference of DEGs, respectively.


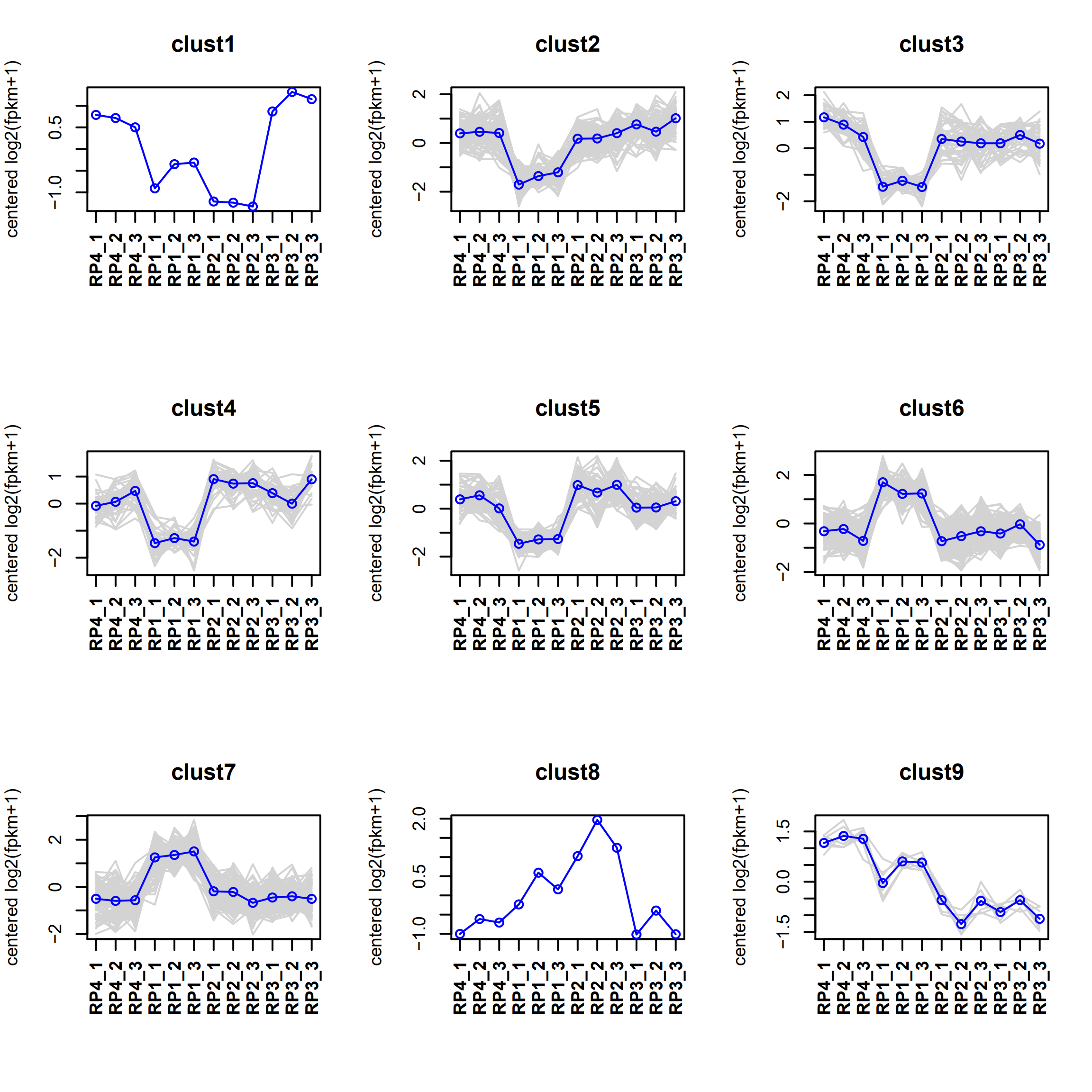


**Figure S2.** 540 DEGs expression trend analysis. The *X*-axis represents four samples of fruit from different periods and the *Y*-axis represents log2(fpkm+1).





**Figure S3.** Quality control (QC) analysis of samples. (A) and (B) QC sample correlation map. The points in each small square represent the ion peaks (metabolites) extracted from QC samples. The *X*-axis and *Y*-axis represent the logarithm of the ion peak signal intensity value. (C) and (D) Relative standard deviation diagram of QC samples. (A), (B) is positive and (C), (D) negative ion mode.





**Figure S4.** OPLS-DA score plots of metabolite profiles of *L. barbarum* fruits. t[1] represents the principal component 1, t[2] represents the principal component 2, the ellipse represents 95% confidence interval. Points of the same color indicate each biological repeat within the group, the distribution of points reflects the degree of difference between and within groups. (A), (C), (E) is positive and (B), (D), (F) negative ion mode, (A), (B) represents the RP1 vs RP2, (C), (D) represents the RP1 vs RP3, (E), (F) represents the RP1 vs RP4.





**Figure S5.** Permutation tests of *L. barbarum* fruit samples. The abscissa in the figure represents the degree of substitution retention, and the ordinate represents the values of R2 and Q2. The green point represents R2, the blue point represents Q2, and the two dashed lines represent the regression lines for R2 and Q2, respectively. (A), (C), (E) is positive and (B), (D), (F) negative ion mode, (A), (B) represents the RP1 vs RP2, (C), (D) represents the RP1 vs RP3, (E), (F) represents the RP1 vs RP4.





**Figure S6.** The relative contents of 8 identical differentially accumulated metabolites in the three comparison groups obtained by metabolome analysis. The *X*-axis represents *L. barbarum* fruit samples and the *Y*-axis represents relative content. Metabolomic analysis is the relative peak area representing the relative content of the detected substance and the values shown are the means ± SD.
